# Protective Effect of *Polygonum orientale* L. Extracts against *Clavibater michiganense* subsp. *sepedonicum*, the Causal Agent of Bacterial Ring Rot of Potato

**DOI:** 10.1371/journal.pone.0068480

**Published:** 2013-07-04

**Authors:** Jin Cai, Shulian Xie, Jia Feng, Feipeng Wang, Qiufeng Xu

**Affiliations:** School of Life Science, Shanxi University, Taiyuan, Shanxi, People’s Republic of China; University of Westminster, United Kingdom

## Abstract

The *Polygonum orientale* L. extracts were investigated for antibacterial activity against *Clavibater michiganense* subsp. *sepedonicum* (Spieckermann & Kotthoff) Davis et al., the causal agent of a serious disease called bacterial ring rot of potato. The results showed that the leaf extracts of *P. orientale* had significantly (*p*<0.05) greater antibacterial activity against *C. michiganense* subsp. *sepedonicum* than root, stem, flower extracts *in vitro*. According to the results of single factor experiments and L_27_3^(13)^ orthogonal experiments, optimum extraction conditions were A_1_B_3_C_1_, extraction time 6 h, temperature 80°C, solid to liquid ratio 1∶10 (g:mL). The highest (*p*<0.05) antibacterial activity was observed when pH was 5, excluding the effect of control. The extracts were stable under ultraviolet (UV). *In vivo* analysis revealed that 50 mg/mL of *P. orientale* leaf extracts was effective in controlling decay. Under field conditions, 50 mg/mL of *P. orientale* leaf extracts also improved growth parameters (whole plant length, shoot length, root length, plant fresh weight, shoot fresh weight, root fresh weight, dry weight, and number of leaves), in the 2010 and 2011 two growing seasons. Further solvent partition assays showed that the most active compounds were in the petroleum ether fractionation. Transmission electron microscopy (TEM) showed drastic ultrastructural changes caused by petroleum ether fractionation, including bacterial deformation, electron-dense particles, formation of vacuoles and lack of cytoplasmic materials. These results indicated that *P. orientale* extracts have strong antibacterial activity against *C. michiganense* subsp. *sepedonicum* and a promising effect in control of bacterial ring rot of potato disease.

## Introduction

*Clavibater michiganense* subsp. *sepedonicum* (Spieckermann & Kotthoff) Davis et al., is a causal agent of a serious disease called bacterial ring rot of potato. This disease has occurred in major potato-growing areas on all continents except Australia [1]–[2], and yield loss in China was up to 60% [3]–[4]. The name of bacterial ring rot of potato originates from the characteristic “ring rot” symptom (destruction of vascular ring) visible after cutting of infected tuber. *C. michiganense* subsp. *sepedonicum* is a highly biotrophic pathogen preferring colonization of the vascular system, particularly the xylem vessels [1]. Colonization of these tissues leads to blocking of the natural transport of water and nutrients followed by wilting of infected leaves and stems. Chemical bactericides e.g. quaternary ammonia, bleach, chlorine dioxide, copper sulfate, potassium permanganate, iodine and phenol groups, are the most commonly used methods for controlling bacterial ring rot of potato [5]–[7]. However, these chemicals have the potential to exert toxic effects on humans and wildlife as well as to cause environment pollution [8]. They also lead to the selection of resistant bacterial populations [9]. In addition, chemical bactericides may not readily be biodegradable and tend to persist for years in environment [10]–[13]. Plants produce a wide variety of physiologically active substances, flavonoids, tannins, alkaloids, saponins sterols, and volatile essential oils [14]. These secondary metabolites are more biodegradable than chemical bactericides and have various functions, including antibacterial activity [15]–[21]. There are few reports available in the literature on the biological prevention and control of *C. michiganense* subsp. *sepedonicum* using plant extracts.

*Polygonum orientale* L. is a fast-growing robust annual herb that is widely distributed in China [22]. It is a traditional Chinese medicinal herb and has been used to treat various diseases, such as fractures, muscle injuries, rheumatism and pain from tissue swelling [23]–[24]. This plant has a porous caudex system, and it can produce large quantities of biomass [25], which may offer a good basis for the production of antibacterial substances. However, no attempts have been made for the management of *C. michiganense* subsp. *sepedonicum* by using *P. orientale* extracts.

The objectives of present study are (1) to evaluate *in vitro* antibacterial activity of *P. orientale* extracts against *C. michiganense* subsp. *sepedonicum*, and to optimize extraction of *P*. *orientale* that can give maximal antibacterial activity. (2) to test the effect of pH and UV on antibacterial activity in *P*. *orientale* extracts. (3) to study *in vivo* effect of *P. orientale* extracts, and growth parameters of potatoes under field conditions. (4) to partition the *P*. *orientale* extracts and determine which fractionation showed the highest antibacterial activity. (5) to determine whether petroleum ether fractionation of *P*. *oriental* extracts lead to cell damage of *C. michiganense* subsp. *sepedonicum* carrying out with TEM.

## Results

### In vitro Antibacterial Activity of P. orientale Extracts against C. michiganense Subsp. Sepedonicum

**Figure 1:**
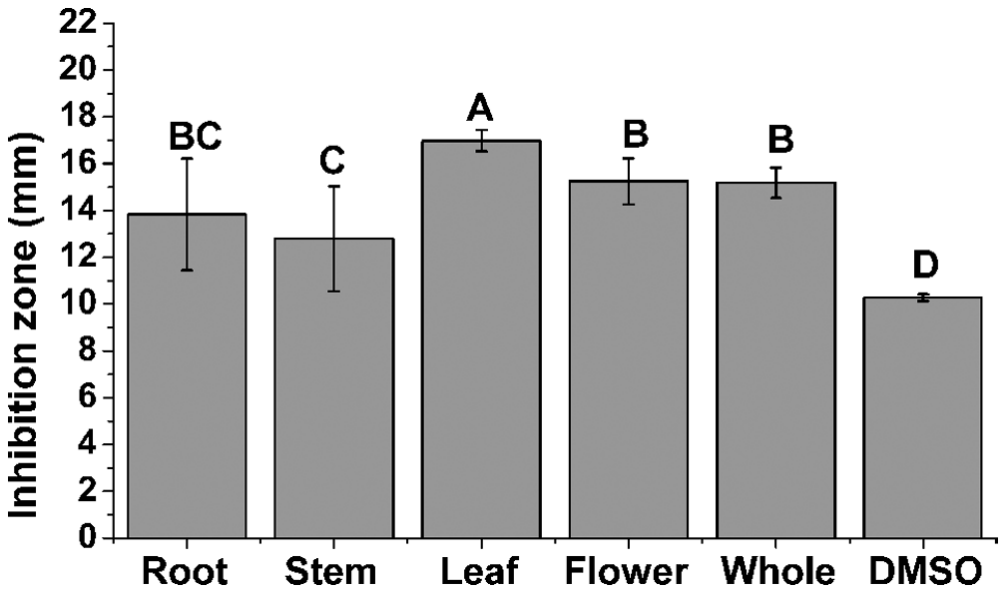
Antibacterial activities of root, stem, leaf, flower and whole plant extracts of *P. orientale*. DMSO was used as control. A negative result was defined as an inhibition zone of 10 mm. Greater than 10 mm indicated positive result of the presence of antibacterial substance. Different letters indicated significant differences (*p*<0.05, ANOVA and Duncan’s multiple range test). Bars represent the means ± standard deviation (S.D.). Each was replicated nine times.

The results presented in Figure 1 showed that root, stem, leaf, flower and whole plant extracts of *P*. *orientale* were all effective in inhibiting the growth of *C. michiganense* subsp. *sepedonicum*, compared to control (*p*<0.05, Figure 1). The leaf extracts of *P*. *orientale* showed the significantly (*p*<0.05) highest antibacterial activity, followed by flower extracts, whole plant extracts, root extracts and stem extracts (Figure 1). Therefore, all subsequent assays were performed with leaf extracts.

### Optimization Study

#### Single factor experiments

**Figure 2:**
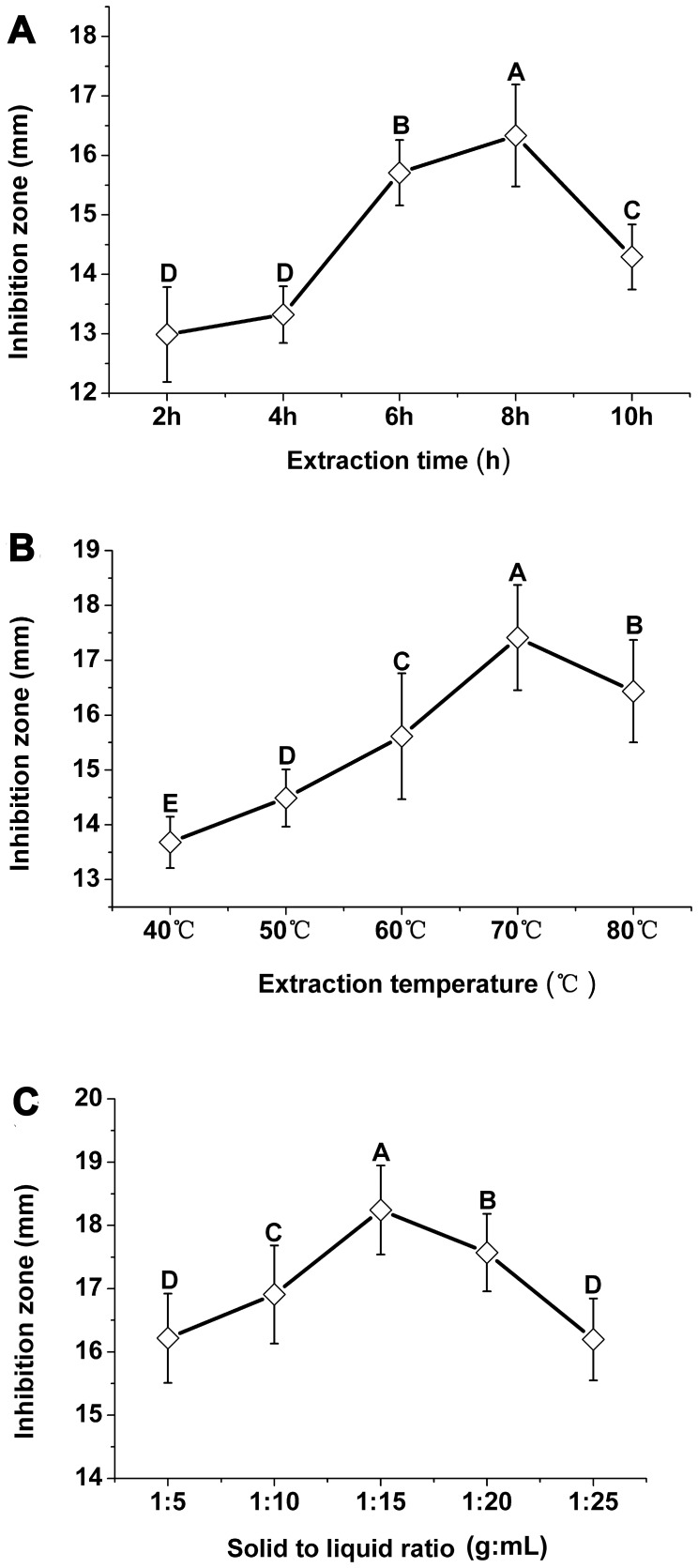
Effect of extraction time (A), extraction temperature (B), and solid to liquid ratio (C). Different letters indicated significant differences (*p*<0.05, ANOVA and Duncan’s multiple range test). Bars represent the means ± standard deviation (S.D.). Each was replicated nine times.

Extraction time (h), extraction temperature (°C), solid to liquid ratio (g:mL) were assessed individually (Figure 2). Figure 2 A depicted the effect of different extraction time on the antibacterial activity in *P*. *orientale* leaf extracts. The antibacterial activity increased with extraction time extended. The highest (*p*<0.05) inhibition zone value was observed at 8 h. Thereafter, antibacterial activity decreased gradually. One-way analysis of variance (ANOVA) shows that the best extraction times with significant (*p*<0.05) difference were Level 3 (6 h), Level 4 (8 h), Level 5 (10 h), and selected them for orthogonal experimental design in Table 1. Increase in temperature led to greater antibacterial activity in extracts (Figure 2 B), and the highest antibacterial activity with significant (*p*<0.05) difference was observed at 70°C. However, increasing temperature did not improve the antibacterial activity at 80°C. ANOVA shows that the best extraction temperatures with significant (*p*<0.05) difference were Level 3 (60°C), Level 4 (70°C), Level 5 (80°C), and selected them for orthogonal experimental design in Table 1. Antibacterial activity of *P*. *orientale* increased with an increasing solid to liquid ratio. Maximum (*p*<0.05) extraction yield of antibacterial substances was achieved at 1∶15 ratio, then antibacterial activity decreased with increasing ratio (Figure 2 C). ANOVA shows that the best solid to liquid ratios with significant (*p*<0.05) difference were Level 2 (1∶10), Level 3 (1∶15), Level 4 (1∶20), and selected them for orthogonal experimental design in Table 1.

**Table 1:** Factors and levels of orthogonal experiment of *P. orientale* leaf extraction.

Levels	Factors		
	Extraction time (A)	Extraction temperature(B)	Solid to liquid ratio(C)
1	6h	60°C	1∶10 (g:mL)
2	8h	70°C	1∶15 (g:mL)
3	10h	80°C	1∶20 (g:mL)

#### Optimization of extraction conditions

**Figure 3:**
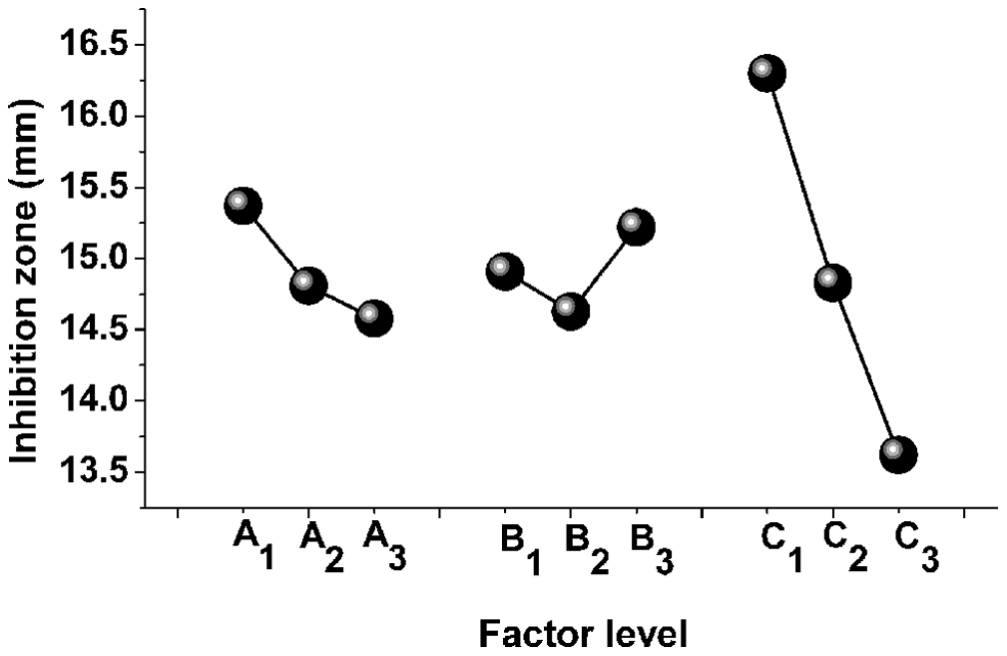
Effect of each parameter on antibacterial activity of *P. orientale*. A, extraction time: A_1_∶6 h, A_2_∶8 h, A_3_∶10 h. B, temperature: B_1_∶60°C, B_2_∶70°C, B_3_∶80°C. C, solid to liquid ratio (g:mL): C_1_∶1:10, C_2_∶1:15, C_3_∶1:20.

Orthogonal experimental design, the main method of fractional factorial design, can effectively screen out key variables by several representative experiments [26]–[27]. From experimental results, it was inferred that antibacterial activity of *P*. *orientale* was influenced by both different factors at different levels and their interactions. The term L_27_ (3^13^) of an orthogonal array implies 27 groups of experiments (Table 2). This array handles up to three factors at three levels each. The subscripts 1, 2, and 3 represent the value of a designed factor at levels 1, 2, and 3 respectively. In other words, these subscripts designate each special trial run of the experiment. For example, in the first row of Table 2 (following the indicated subscripts), the factor level of factor A (which is assigned to the first column of the array) is 1, and the level of factors B and C are 1 as well. The first trial run of this experiment will be designed as a level set {1, 1, 1} for factors A, B, C according to Table 1. Therefore the first experiment was carried under extraction time 6 h, temperature 60°C, solid to liquid ratio 1∶10 (g:mL) conditions. The other experiments would perform in the same way, and the experimental results of the orthogonal design were shown in Table 2. Factors that influence antibacterial activity of *P*. *orientale* were listed in a decreasing order as follow: C>A>B (Table 2). The individual levels within each factor were ranked as Figure 3: A: 1>2>3; B: 3>1>2; C: 1>2>3.

**Table 2:** Orthogonal experiment L_27_ (3^13^) and intuitive analysis.

ExperimentNO.	Factors			Inhibition zone (mm)
	Extractiontime (A)	Extraction temperature (B)	Solid toliquid ratio (C)	*C.michiganense* subsp. *sepedonicum* a
1	1 (6h)	1 (60°C)	1 (1∶10, g:mL)	15.57±2.53
2	1 (6h)	1 (60°C)	2 (1∶15, g:mL)	14.11±1.63
3	1 (6h)	1 (60°C)	3 (1∶20, g:mL)	15±1.91
4	1 (6h)	2 (70°C)	1 (1∶10, g:mL)	16.97±3.01
5	1 (6h)	2 (70°C)	2 (1∶15, g:mL)	14.38±1.72
6	1 (6h)	2 (70°C)	3 (1∶20, g:mL)	13.85±1.11
7	1 (6h)	3 (80°C)	1 (1∶10, g:mL)	17.35±1.32
8	1 (6h)	3 (80°C)	2 (1∶15, g:mL)	16.16±0.96
9	1 (6h)	3 (80°C)	3 (1∶20, g:mL)	14.97±0.92
10	2 (8h)	1 (60°C)	1 (1∶10, g:mL)	15.62±1.45
11	2 (8h)	1 (60°C)	2 (1∶15, g:mL)	15.16±3.08
12	2 (8h)	1 (60°C)	3 (1∶20, g:mL)	12.92±1.53
13	2 (8h)	2 (70°C)	1 (1∶10; g:mL)	15.95±2.83
14	2 (8h)	2 (70°C)	2 (1∶15, g:mL)	15.1±1.94
15	2 (8h)	2 (70°C)	3 (1∶20, g:mL)	11.45±1.90
16	2 (8h)	3 (80°C)	1 (1∶10, g:mL)	16.33±2.33
17	2 (8h)	3 (80°C)	2 (1∶15, g:mL)	14.87±1.07
18	2 (8h)	3 (80°C)	3 (1∶20, g:mL)	15.86±3.42
19	3 (10h)	1 (60°C)	1 (1∶10, g:mL)	18.49±0.77
20	3 (10h)	1 (60°C)	2 (1∶15, g:mL)	14.06±1.63
21	3 (10h)	1 (60°C)	3 (1∶20, g:mL)	13.24±1.39
22	3 (10h)	2 (70°C)	1 (1∶10, g:mL)	15.4±1.53
23	3 (10h)	2 (70°C)	2 (1∶15, g:mL)	15.36±2.66
24	3 (10h)	2 (70°C)	3 (1∶20, g:mL)	13.17±2.48
25	3 (10h)	3 (80°C)	1 (1∶10, g:mL)	15.06±1.80
26	3 (10h)	3 (80°C)	2 (1∶15, g:mL)	14.3±1.62
27	3 (10h)	3 (80°C)	3 (1∶20, g:mL)	12.1±0.97
*K*_1*j*_ b	15.37	14.91	16.30	∑402.8
*K*_2*j*_	14.81	14.63	14.83	
*K*_3*j*_	14.58	15.22	13.62	
*R* c	0.79	0.59	2.68	
*O* d	A_1_	B_3_	C_1_	

a DMSO was used as control. A negative result was defined as an inhibition zone of 10 mm. Greater than 10 mm indicated positive result of the presence of antibacterial substance (S.D.). Each value was mean and standard deviation of four replications.

b K*_ij_* = (1/9) ∑ mean inhibition zone at factor *j* (*j* = A, B, C).

c R*_ij_* = max { K*_ij_* } − min { K*_ij_* }, *j* and *i* mean factor and setting level here, respectively.

d O means the optimum condition. The optimum combination of conditions is A_1_B_3_C_1_.

**Figure 4:**
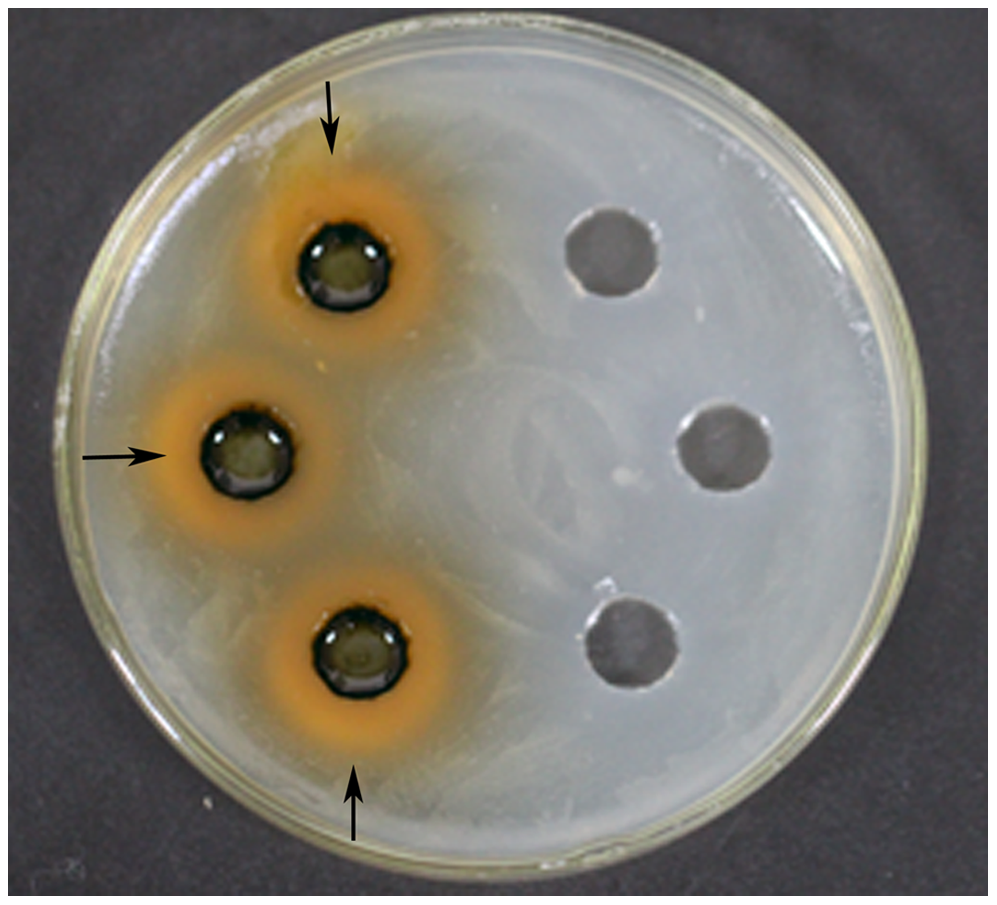
Antibacterial activity of *P. orientale* leaf extracts against *C. michiganense* subsp. *sepedonicum.* Using optimum combination of extraction conditions (extraction time 6 h, temperature 80°C, solid to liquid ratio (g:mL) 1∶10, left). DMSO was used as control (right).

Because interactions between factors are complex, only low-order interactions were analyzed while high-order (three-, four-, and five-order) interactions were neglected. Table 3 summarize the analysis of variance (ANOVA) of factors and their second-order interactions that affect antibacterial activity. In Table 3, the term “interaction”, indicated by inserting the “×” symbol between the two interacting factors, is used to describe the condition in which the effect of one factor’s influence upon the result is dependent on the condition of the other factor. *F-*ratio is defined as *F* = MSF/MSE, where MSF and MSE represent respectively mean square of factors or interactions, mean square of errors. *df*, SS and MS respectively represent degree of freedom, sum of squares and mean square. If the calculated value *F* is greater than critical value *F*_α_ [e.g. *F*_0.01_(2,8) = 8.65], then that factor or interaction is statistically significant. In Table 3, if significant level α = 0.01, then C (Solid to liquid ratio) was statistically significant factor that affect antibacterial activity of *P*. *orientale.* Therefore, factor C was regarded as dependent factor in extraction of antibacterial substance. A (Extraction time), B (Temperature) and interactions A×B, A×C, B×C were regarded as independent factors and interactions. Optimum values of these factors for extraction of antibacterial substance from *P*. *orientale* were A_1_B_3_C_1_, extraction time 6 h, temperature 80°C, solid to liquid ratio 1∶10 (Table 2). Through optimization test, the inhibition zone was up to 19.54 mm (Figure 4).

**Table 3:** Results of variance (ANOVA) analysis.

Source	SS	*df*	MS	*F* a	Significance^b^
A (Extraction time)	3.03	2	1.52	1.02	
B (Extraction temperature)	1.60	2	0.80	0.54	
C (Solid to liquid ratio)	32.58	2	16.29	10.93	**
A×B (Interaction of extraction time and extraction temperature )	8.09	4	2.02	1.36	
A×C (Interaction of extraction time and solid to liquid ratio)	2.86	4	0.72	0.48	
B×C(Interaction of extraction temperature and solid to liquid ratio)	2.80	4	0.70	0.47	
Error	11.90	8	1.49		
Total	62.86	26			

a Significant parameter, *F*_0.05_ (2, 8) = 4.46, *F*_0.05_ (4, 8) = 3.84, *F*_0.01_ (2, 8) = 8.65, *F*_0.01_ (4, 8) = 7.01.

b ** indicated more significant difference.

### Effect of pH and UV on the Antibacterial Activity

**Figure 5:**
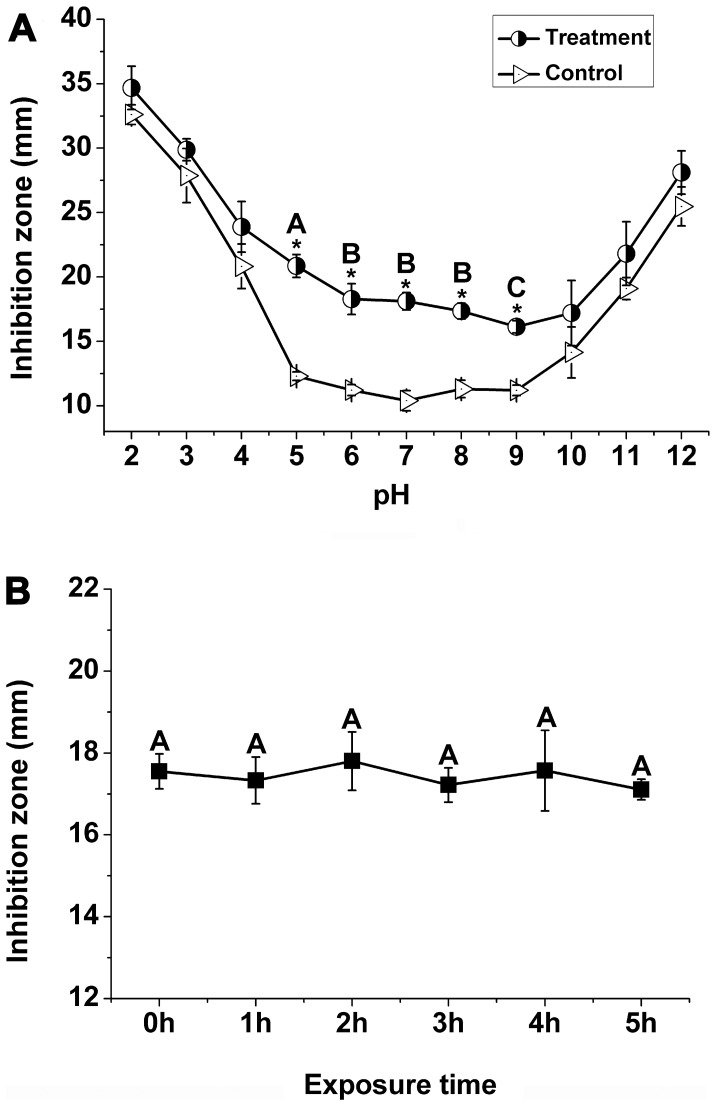
Effect of pH (A) and UV (B) on antibacterial activity in *P. orientale* leaf extracts. For pH effect, *t*-test was carried out to determine significant (*p*<0.05) differences between test sets and control sets. * indicated significant differences. ANOVA was carried out to determine significant (*p*<0.05) differences between test sets at different pH values ranging from 5 to 9. Different letters indicated significant differences (*p*<0.05). Bars represent the means ± standard deviation (S.D.). Each was replicated four times.

As shown in Figure 5 A, there were no statistically significant (*p*<0.05) differences between test sets and control sets in the range from pH2 to pH4, and pH10 to pH12. The result implied that, antibacterial activity was enhanced under strongly acidic and alkaline conditions (pH2 to pH4, and pH10 to pH12), but this might be the role of acid and alkali, rather than *P. orientale* leaf extracts. Based on *t*-test, we got the result that there were statistically significant (*p*<0.05) differences between test sets and control sets in the range from pH 5 to pH 9. In other words, antibacterial activity of *P. orientale* leaf extracts was not impacted by the control in the range from pH 5 to pH 9. In order to exclude the effect of control, we only compared the antibacterial activity of test sets when pH values were between 5 and 9, based on ANOVA. The data showed that maximum efficiency of antibacterial activity was observed when pH was 5 (*p*<0.05), but it decreased rapidly when pH values were between 6 and 9.

To test the UV stability of *P. orientale* extracts, we investigated the antibacterial activities of different treatments. There were no statistically significant (*p*<0.05) differences between samples that were exposed to UV light for different times (Figure 5 B), implying that *P. orientale* extracts were not impacted by exposure to UV light.

### Protective Effects of *P. orientale* Leaf Extracts

**Figure 6:**
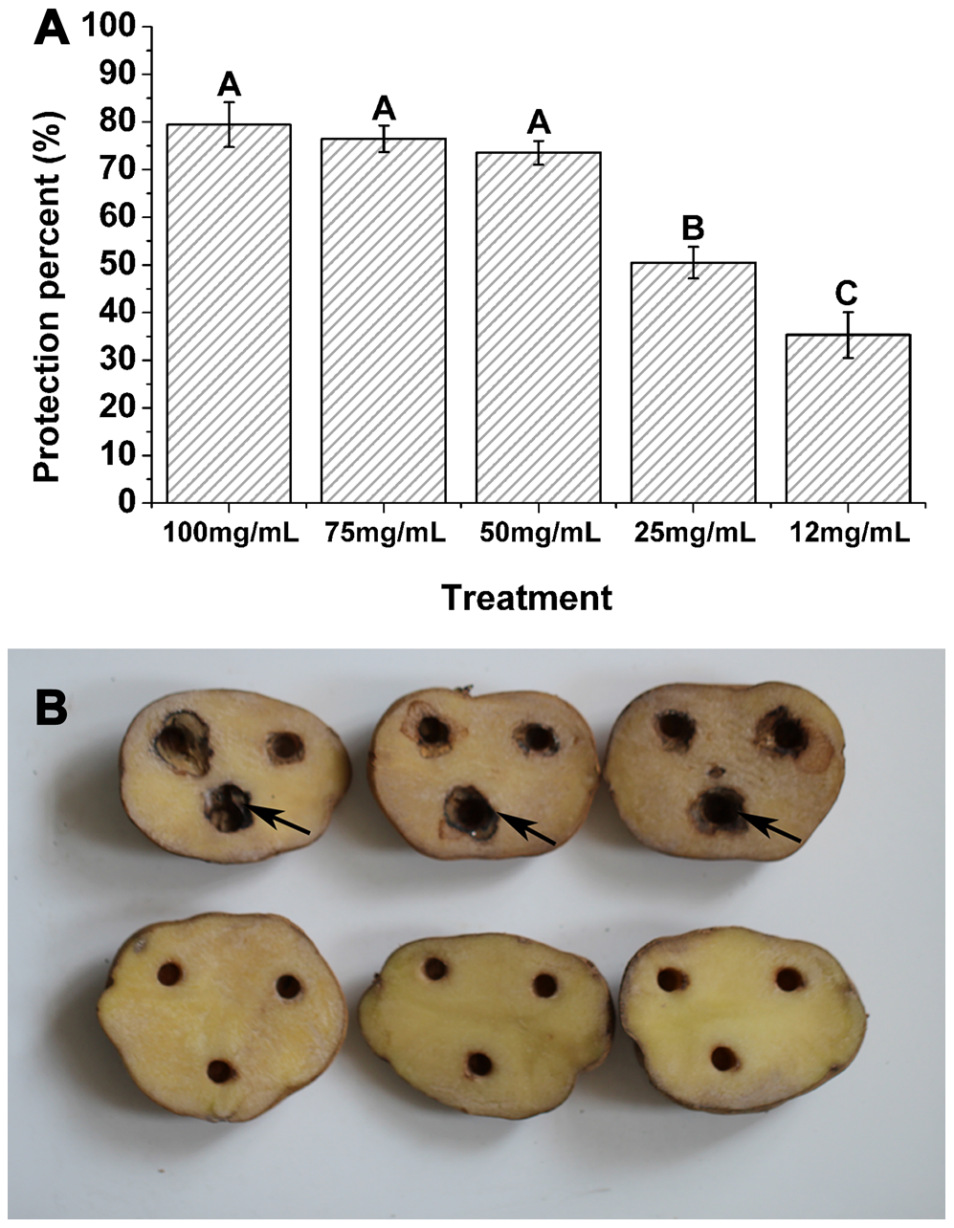
*In vivo* inhibition analysis of *P. orientale* leaf extracts against *C. michiganense* subsp. *sepedonicum*. Protection percent treated with 100 mg/mL, 75 mg/mL, 50 mg/mL, 25 mg/mL, 12 mg/mL of *P. orientale* leaf extracts (A). Different letters indicated significant differences (*p*<0.05, ANOVA and Duncan’s multiple range test). Bars represent the means ± standard deviation (S.D.). Each was replicated four times. Control, the number of potatoes was 51, which treated with distilled water showing disease symptom. Tuber lesions caused by bacteria (B). Prior to incubation, water solution of *P. orientale* leaf extracts at concentration of 50 mg/mL was put into the holes of the treatment group (bottom), whereas sterilized water was put into the control (top). Then, all tubers were inoculated with *C. michiganense* subsp. *sepedonicum*, and incubated for three days. Tubers treated with water solution of *P. orientale* leaf extracts at concentration of 50 mg/mL were uninfected (bottom). By contrast, the tubers treated with sterilized water in the control group were severely infected by *C. michiganense* subsp. *sepedonicum* and manifested aggressive lesions (top). The resulting lesions were highlighted with arrowheads.

To determine whether *P. orientale* leaf extracts exerted *in vivo* inhibition against *C. michiganense* subsp. *sepedonicum*, an inoculation experiment was performed. As shown in Figure 6 A, water solution of *P. orientale* leaf extracts had 35.29% protective effect at a lower concentration of 12 mg/mL, 50.49% protective effect at 25 mg/mL. At 50 mg/mL, 75 mg/mL and 100 mg/mL concentrations, the protective effect reached 73.55%, 76.47% and 79.44%. However, there were no statistically significant (*p*<0.05) differences between 50 mg/mL, 75 mg/mL and 100 mg/mL concentrations, and there were statistically significant (*p*<0.05) differences between 12 mg/mL, 25 mg/mL and 50 mg/mL concentrations, based on ANOVA. These results clearly demonstrated strong *in vivo* inhibition against *C. michiganense* subsp. *sepedonicum* by *P. orientale* leaf extracts. Figure 6 B showed tuber lesions caused by bacteria.

### Plant Growth Parameters under Field Conditions

In the present study, growth parameters were recorded after 8 weeks from date of planting during the 2010 and 2011 two growing seasons. Data in Table 4 showed that *P*. *orientale* leaf extracts at different concentrations were able to increase the whole plant length and shoot length significantly (*p*<0.05), compared to negative control, during both growing seasons. 50 mg/mL of extracts increased root length, plant fresh weight, shoot fresh weight and dry weight significantly (*p*<0.05) in both seasons, compared to negative control. Thus, extracts at 25 mg/mL and 12 mg/mL concentrations were not effective in increasing these parameters. The root fresh weight showed no significantly differences between the *P*. *orientale* leaf extract treatments and the negative control in 2010 season. Thus, 50 mg/mL of extracts increased root fresh weight significantly (*p*<0.05) compared to 25 mg/mL and 12 mg/mL of extracts and negative control, in 2011 season. Extracts at 50 mg/mL and 25 mg/mL concentrations increased the final number of leaves significantly (*p*<0.05), in 2010 season. In contrast, 50 mg/mL, 25 mg/mL and 12 mg/mL of extracts were all effective in increasing the final number of leaves, in 2011 season.

**Table 4:** Effect of different concentrations (50 mg/mL, 25 mg/mL and 12 mg/mL) of *P. orientale* leaf extracts on different growth parameters of potato under field conditions during the 2010 and 2011 growing seasons.

Treatments	Whole plantlength (cm)	ShootLength (cm)	Rootlength (cm)	Plant freshweight (g)	Shoot freshweight (g)	Root freshweight (g)	Dry weight (g)	Number of leaves
Season 2010								
50 mg/mL ofextracts	45.40±5.46A	24.60±1.14A	20.80±3.41A	38.58±4.38AB	32.08±3.92AB	6.50±0.82A	6.00±1.58AB	50.33±2.52A
25 mg/mL ofextracts	39.20±4.49A	23.40±4.72A	15.80±2.49AB	31.28±9.37BC	25.90±9.73BC	5.38±0.87A	4.48±0.90BC	49.00±1.73A
12 mg/mL ofextracts	38.60±1.14A	22.10±3.44A	16.50±3.81AB	23.60±4.10C	18.84±2.47BC	4.76±2.11A	3.92±0.87C	38.33±8.02B
50 mg/L of copper sulfate(positive control)	45.60±2.00A	24.00±1.48A	21.60±1.79A	46.58±7.35A	40.68±10.45A	5.90±1.65A	6.72±1.31A	56.67±9.02A
Untreated(negativecontrol)	29.00±3.15B	15.20±4.63B	13.80±3.29B	19.70±3.00C	14.68±4.31C	5.02±0.34A	3.54±0.43C	28.33±1.53B
Season 2011								
50 mg/mL ofextracts	49.60±2.16A	27.57±0.38A	22.03±1.82A	45.13±3.00A	37.87±4.00A	7.26±0.98A	7.26±0.70A	55.33±4.16AB
25 mg/mL ofextracts	46.00±4.00A	27.03±1.10A	18.97±3.10AB	31.82±2.21B	25.91±1.61B	5.91±0.60B	4.91±0.69B	53.67±5.51B
12 mg/mL ofextracts	45.00±2.00A	26.47±1.03A	18.53±3.01AB	31.05±2.63B	25.05±2.75B	6.00±0.25B	4.62±0.46B	53.33±3.06B
50 mg/L of copper sulfate(positive control)	48.00±2.00A	25.73±1.60A	22.27±0.42A	45.96±3.30A	38.45±3.92A	7.51±0.78A	6.77±0.33A	61.33±3.06A
Untreated (negativecontrol)	30.00±1.55B	14.80±1.71B	15.20±0.30B	27.13±1.59B	22.03±1.83B	5.10±0.36B	4.01±0.54B	30.67±2.52C

All data are average of three replications. Each value was the mean with standard deviation (S.D.). Different letters indicated significant differences (*p*<0.05, ANOVA and Duncan’s multiple range test). Untreated potato tubers were used as the negative control, and 50 mg/L of copper sulfate was used as the positive control.

### Partition of the Ethanol Extracts

**Figure 7:**
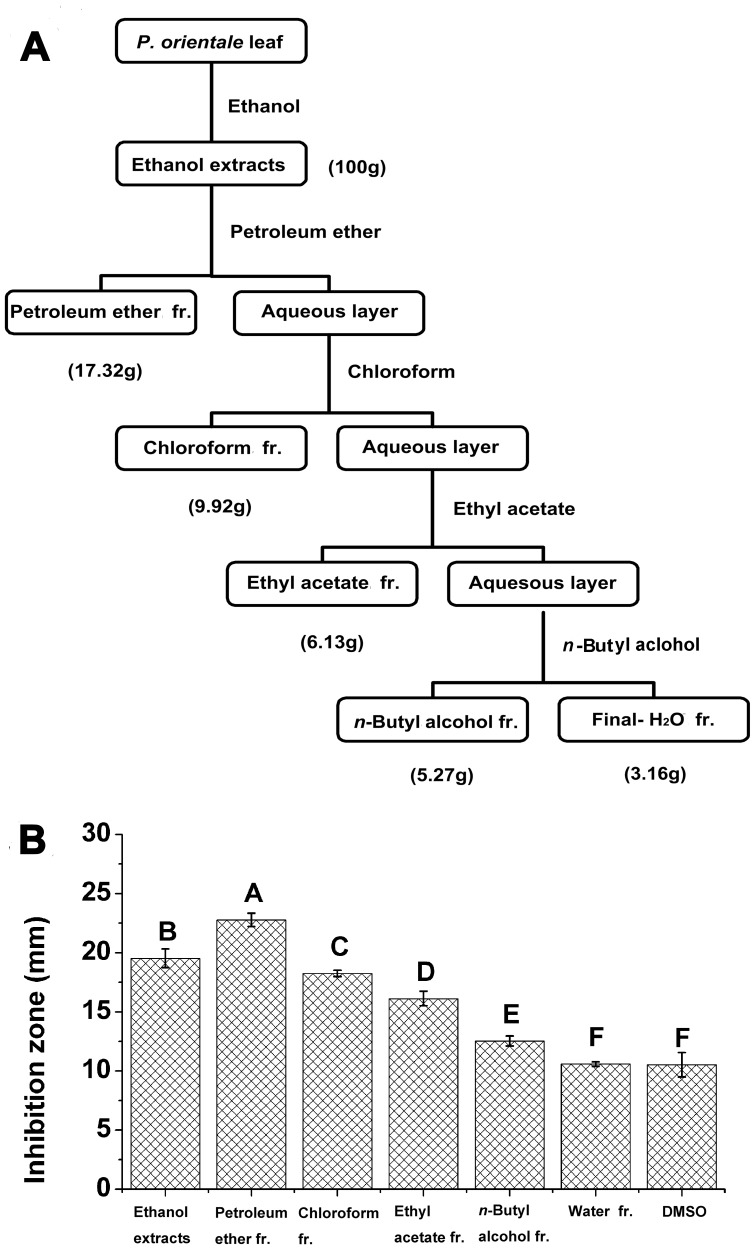
Fractionation charts of *P. orientale* extracts (A), and antibacterial activities of fractionations (B). Fr. means fractionation. Different letters indicated significant differences (p<0.05, ANOVA and Duncan’s multiple range test). Bars represent the means ± standard deviation (S.D.). Each was replicated four times.

The ethanol extracts were partitioned with four solvents and the antibacterial activity was measured in five partitions (Figure7). The amount of petroleum ether fractionation (17.32 g) was the largest among the five fractionations, compared with 9.92 g in chloroform fractionation, 6.13 g in ethyl acetate fractionation, 5.27 g in *n*-butyl alcohol fractionation and 3.16 g in water fractionation (Figure 7 A). At a concentration of 1 mg/mL, the petroleum ether fractionation showed the highest (*p*<0.05) antibacterial activity, followed by ethanol extracts (positive control), chloroform fractionation, ethyl acetate fractionation, *n*-butyl alcohol fractionation, water fractionation and DMSO (negative control, Figure7 B). These results suggested that the petroleum ether fractionation was the most efficient in inhibiting the *C. michiganense* subsp. *sepedonicum*.

### Observation of Interior Damage

**Figure 8:**
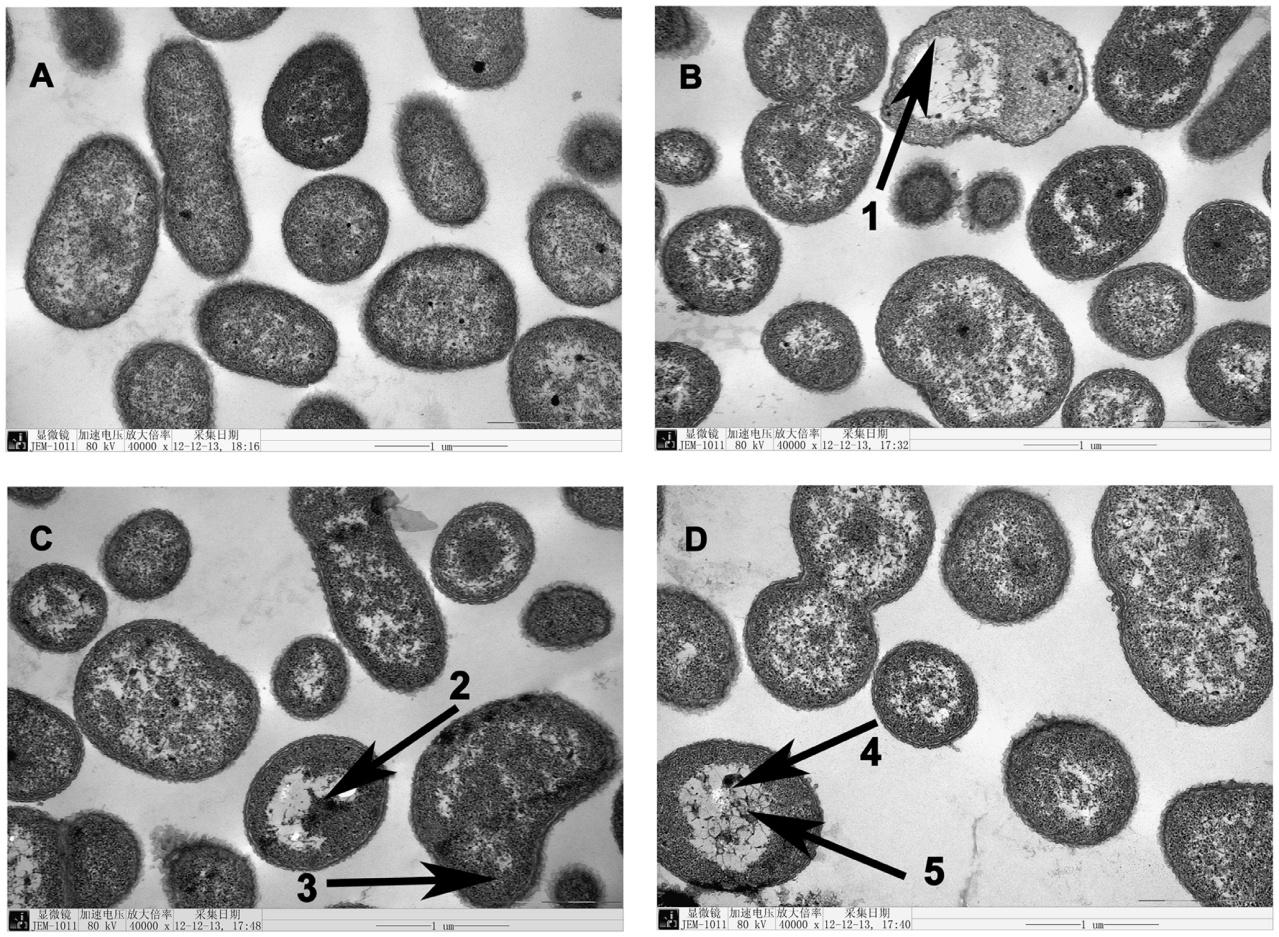
Transmission electron micrographs of *C. michiganense* subsp. *sepedonicum* cells. Untreated (A, 40.000x). Treated with the petroleum ether fractionation of *P. orientale* extracts (B,C,D, 40.000x). Malformation of cells (1, 3). Formation of vacuoles and loss of cytosol structure and contents inside the cells (1, 2, 5). The cytoplasm was coagulated (4).

The influence of petroleum ether fractionation from *P. oriental* extracts on the cell morphology of *C. michiganense* subsp. *sepedonicum* was investigated by TEM. Untreated cells showed no changes in cell morphology after eight hours. Cell showed a typical cell wall, cytoplasmic membrane, periplasmic space, and cytoplasmic content (Figure 8 A). In contrast, *C. michiganense* subsp. *sepedonicum* treated with petroleum ether fractionation (0.05 mg/mL) exhibited a wide range of abnormalities (Figure 8 B–D). Compared with undamaged cells, it was easy to find small vacuoles inside the cells (Figure 8 B–D). Some cells showed formation of big vacuoles (pure-white regions of bacteria) and lack of cytoplasmic material (Figure 8 (1, 2, 5)), others showed bacteria misshapen (Figure 8 (1, 3)). In addition, electron-dense particles were also observed in damaged bacterial cell (Figure 8 (4)).

## Discussion

The exploitation of plant products for the management of plant diseases has made significant progress due to its readily available nature, easy biodegradability, non-phytotoxicity [28]. Recently, several studies have been reported to use plant extracts in controlling plant diseases [29]–[30]. In the present study, we evaluated the antibacterial activity in *P. orientale* extracts against *C. michiganense* subsp. *sepedonicum*. Among root, stem, leaf, flower and whole plant extracts of *P*. *orientale*, the leaf extracts showed significantly (*p*<0.05) highest antibacterial activity (Figure 1). This demonstrated that *P. orientale* played an important role in biological control of *C. michiganense* subsp. *sepedonicum*, the causal agent of bacterial ring rot of potato. In addition, this is the first report of *P. orientale* as a potential agent against *C. michiganense* subsp. *sepedonicum*.

Solvent played a key role in extraction of antibacterial substances from *P*. *orientale*. In this study, we used ethanol. Ethanol has high polarity index, dielectric constant and cohesive energy, as compared with other solvents, which provides strong bounding between solvent molecules and compounds from the solutes, causing their dissolution [31]. In addition, ethanol has several advantages such as low toxicity, economical, and lower boiling point [31]. There are many factors affecting the extraction, among them, extraction time (A), extraction temperature (B), and solid to liquid ratio (C) are key factors. Single factor experiment was performed by one factor varied with different levels while other factors being fixed. Shorter extraction times would result in incomplete extraction. Longer extraction times would lead to waste of time and energy, and antibacterial components might be decomposed (Figure 2 A) [32]. Increasing temperature enhanced diffusivity and thus the yield of antibacterial activity in extracts was increased with higher temperature [33]. When temperature was too high, ethanol volatilization was accelerated and the solid to liquid ratio was lowed, and thus the yield of antibacterial activity was decreased (Figure 2 B). When the solid to liquid ratio was too low, the contact between antibacterial substance and solvent was not sufficient enough, and it was not conducive to extract maximal amount of antibacterial substances. When the solid to liquid ratio was too high, concentration time would be long and antibacterial components might be decomposed (Figure 2 C) [34].

The orthogonal experimental design was used to study optimization of parameters for efficient extraction of antibacterial substances from *P*. *orientale*. The advantage of orthogonal experimental design is that its economical for characterizing a complicated process in fewer experiments. However, it requires a specialized experimental design to properly set up the test and specialized statistics to analyze data[35]–[37]. The results (Table 2, Table3) revealed that factor C (Solid to liquid ratio) had significant effect on the antibacterial activity, while the other factors and interactions were not identified as significant factors and interactions under the selected conditions based on ANOVA. We concluded that solid to liquid ratio was the major factor affecting *P*. *orientale* extraction. Thus, we should pay more attention to the factor in extraction. The optimum extraction conditions for *P*. *orientale* were defined as below: extraction time: 6 h, temperature: 80°C, solid to liquid ratio: 1∶10 (g:mL). Compared with conventional extraction conditions, our optimum extraction conditions in this study are economic, convenient and efficient [38]. Further, this extraction method meets the actual needs and is also compliant with environmental regulations.

Environmental factors often influence the efficacy of bactericides [39]. In this study, we tested whether pH and UV could influence the efficacy of *P. orientale* leaf extracts. Data (Figure 5 A) showed that the highest (*p*<0.05) antibacterial activity was observed when pH was 5, excluding the effect of control. This indicated that either organic acids or other pH-dependent antibacterial compounds were responsible for the antibacterial effect [40]. These results indicated that *P. orientale* leaf extracts would be best used when pH was 5. As exposure time changed, no statistically significant (*p*<0.05) differences were observed between different UV treatments (Figure 5 B). The results showed that extracts were stable following exposure to UV.

Although *in vitro* test of plant extracts is an important first step in selecting plants with potential antibacterial activity against plant pathogens, *in vivo* test is reproducible [41]. Results (Figure 6) obtained from the *in vivo* study indicated that *P. orientale* leaf extracts contained strong antibacterial activity against *C. michiganense* subsp. *sepedonicum in vivo*. Our finding of the protective effect of *P. orientale* leaf extracts provides evidence that the antibacterial potentiality of *P. orientale* leaf extracts can be used as an alternative to bactericides.

*P*. *orientale* leaf extracts were tested at different concentrations for increasing the plant growth under field condition in the 2010 and 2011 two growing seasons. All the treatments were significant (*p*<0.05) for increases in whole plant length and shoot length promotion, compared to negative control, during both growing seasons (Table 4). A clear correlation was observed between plant growth parameters and concentrations of extraction. Among different treatments, 50 mg/mL had the best performance for most of the parameters assessed, in both 2010 and 2011 growing seasons (Table 4). Our results indicated that application of *P. orientale* leaf extracts was effective in increasing the plant growth under field condition.

The results of partition showed that the antibacterial material exists largely in the petroleum ether phase. Su et al, (2012) [42] had identified chemical composition of petroleum ether fractionation from *P*. *orientale* ethanol extracts by using GC-MS method, for anti-tumor research. Therefore, we did not repeat this experiment. As the results, forty eight components were identified. The major components were separated into six classes, including nexplanon (18.31%), alkane (1.62%), ester (10.95%), alcohol (9.65%), organic acids (10.55%) and ketone (7.36%). The mechanism of action of these compounds is not fully understood. But among these compounds, ethyl laurate, hexadecanoic acid, ethyl oleate, hexadecane, clionasterol, eicosanoic acid and stigmasterol are known to exhibit antibacterial activity [43]–[54]. On the other hand, it is speculated that cell membrane disruption by lipophilic compounds (nexplanon, alkane, ester and ketone) may be involved [55]. Because the highly lipophilic compounds easily pass through cell membranes to induce biological responses [56]. Third, it is possible that the active components might be involved in some type of synergism in antibacterial activity with lipophilic compounds [57].

To investigate possible changes in cell morphology, TEM was applied. *C. michiganense* subsp. *sepedonicum*, treated with petroleum ether fractionation of *P. oriental* extracts, showed electron-dense particles, bacteria misshapen, formation of vacuoles and lack of cytoplasmic materials (Figure 8). These results indicated that compounds from the petroleum ether fractionation had penetrated the cell walls and cell membranes, and interacted with cellular contents. Considering the constituents of petroleum ether fractionation from *P. oriental* extracts, it was most likely that antibacterial activity was not attributable to one specific mechanism, since there were several targets in the cell. The components of petroleum ether fractionation included nexplanon, alkane, ester, alcohol, organic acids and ketone. Lipophilic compounds had the ability to interact with hydrophobic structures, like bacterial membranes [58]. It was speculated that lipophilic compounds disrupted the cytoplasmic membrane of *C. michiganense* subsp. *sepedonicum*, thereby causing leakage of the bacterial cell content. Furthermore, the dysfunction and disruption of the membrane, interference with the energy generation system in cell, and enzyme inhibition preventing substrate utilization for energy production might also lead to the death of bacterial cells [59]–[61]. In TEM, the appearance of electron-dense particles might be result from several possible events. The petroleum ether fractionation contained antibacterial substances, such as ethyl laurate, hexadecanoic acid, ethyl oleate, hexadecane, clionasterol, eicosanoic acid and stigmasterol. These substances might be due to the precipitation of abnormal proteins, and we could see protein aggregation as electron-dense particles in the TEM. Based on the present research, schematic model for proposed mechanism was described as follows. Lipophilic materials in petroleum ether fractionation made a break through the outer membrane firstly, causing the leakage of cellular contents. Secondly, antibacterial materials in petroleum ether fractionation entered the inner membrane, thus inhibiting respiration and growth of cells. Simultaneously, antibacterial materials could affect some proteins, resulting in cell decomposition and death eventually.

### Conclusion

In conclusion, *P. oriental* extracts consistently showed significant antibacterial activity against *C. michiganense* subsp. *sepedonicum* in *in vitro*, *in vivo* and in field experiments, respectively. The optimum extraction conditions were investigated using single factor experimental design and L_27_3^(13)^ orthogonal experimental design. The maximum efficiency of antibacterial activity was observed when pH was 5. The extracts were relatively stable when exposed to UV radiation. From partition study, it has become clear that petroleum ether fractionation of *P*. *orientale* extracts showed the greatest potential to inhibit the growth of *C. michiganense* subsp. *sepedonicum*. TEM investigated the possible mechanism of petroleum ether fractionation against *C. michiganense* subsp. *sepedonicum*. Results of TEM revealed that petroleum ether fractionation of *P*. *orientale* extracts caused cytoplasm coagulated in cell, bacterial misshapen, formation of vacuoles and lack of cytoplasmic material. These findings indicate that *P. oriental* extracts have a great potential for biological control of *C. michiganense* subsp. *sepedonicum*. The antibacterial activity of the agent against *C. michiganense* subsp. *sepedonicum* should be applied in the field for potato protection. The agent offers a safe alternative to synthetic bactericide.

## Materials and Methods

### Plant Material and Pathogen

*Polygonum orientale* L. was collected from wetland of Fenhe River in Taiyuan section, Shanxi Province, China, in July 2008. The collection of plant is not need specific permissions, and the field studies did not involve endangered or protected species. Taxonomic identification was performed in our lab. *Clavibater michiganense* subsp. *sepedonicum* (Spieckermann & Kotthoff) Davis et al. (ATCC 33113) was provided by Chinese Academy of Agricultural Science. Potato cultivar ‘Jinhan*-*1′ was obtained from a local seed agency.

### Preparation of Plant Extracts

The roots, stems, leaves and flowers of *P*. *orientale* were cut into small pieces (2–4 cm) respectively. Each was washed several times with running tap water, then with sterile water, and dried at room temperature for 15 days [62]. Dry materials were ground to fine powders in a grinder. Then 100 g of each powder was blended in 1 L of ethanol at room for 24 h [63]. The extracts were concentrated to dryness using a rotary evaporator after filtrating.

### In vitro Assays

Determination of antibacterial activity was accomplished by agar diffusion method (ADM) [64]. Each residue was dissolved in DMSO (dimethyl sulphoxide) to give a final concentration of 1 mg/mL. Fresh strain (18–24 h old) grown in nutrient broth was used for the studies. The medium contained 1 L distilled water, 5 g beef extract, 10 g pepton, 5 g sodium chloride, 20 g agar. The agar surface was perforated with 10 mm diameter holes, aseptically cut and filled with 200 µL of each sample. The DMSO was used as control since it does not inhibit microorganism growth [65]. After the diffusion of the solution in each hole, the plates were inverted and incubated at 28°C for 24 h. Antibacterial activity was determined by measuring the radius of the inhibition zone around the hole. Each treatment was replicated nine times.

### Optimization of Extraction Condition

#### Single factor experiments

The three factors including extraction time (h), extraction temperature (°C), solid to liquid ratio (g:mL) could affect extraction efficiency. Single factor experiments were applied to decide appropriate levels. For each single factor, five different levels were designed, with other factors being kept constant. For each experiment, 100 g of *P*. *orientale* leaf sample was added to corresponding volume of ethanol and extracted as described in Table 5. Then, antibacterial activity of extracts (1 mg/mL) from each sample was analyzed by ADM to choose three reasonable levels of three factors for orthogonal experimental design. Each treatment was replicated nine times.

**Table 5:** Single factor experiment design.

Factors	Conditions	Levels				
		1	2	3	4	5
Extraction time (h)	Temperature 80°C	2	4	6	8	10
	Solid to liquid ratio 1∶5					
Temperature (°C )	Extraction time 8h	40	50	60	70	80
	Solid to liquid ratio 1∶5					
Solid to liquid ratio (g:mL)	Extraction time 8h	1∶5	1∶10	1∶15	1∶20	1∶25
	Temperature 80°C					

#### Orthogonal experimental design

On the basis of single factor experiments, three levels of three factors were selected as described in Table 1. Then orthogonal array L_27_(3^13^) matrix was used to determine the optimum extraction conditions of antibacterial substances from *P*. *orientale*, with the consideration of the interactions between the parameters [66]–[67]. For each experiment, 100 g of *P*. *orientale* leaf sample was added to corresponding volume of ethanol and extracted as described in (Table 2). Four replicates were used for each extraction. Then, antibacterial activity of extracts (1 mg/mL) from each sample was analyzed by ADM. Each treatment was replicated four times. ANOVA were performed by using SPSS software package (version 17.0) to identify significant extraction factor and interaction between samples.

### pH and UV Stability Assays

The effect of pH on antibacterial activity in *P*. *orientale* leaf extracts was examined by pH stability assays [68]–[69]. Tests were conducted in two sets: test sets of *P*. *orientale* leaf extracts were adjusted with 5 M NaOH or 5 M HCl to different pH values ranging from 2 to 12. The control sets were prepared using the same method with DMSO except that no *P*. *orientale* leaf extract was added. To test the impact of UV, *P*. *orientale* leaf extracts were incubated under UV light (256rim, 6W, 5 cm) for a period ranging from 1 h to 5 h. Then, antibacterial activity of extracts (1 mg/mL) was analyzed by ADM. Each treatment was replicated four times.

### In vivo Assays

The dried powder of *P*. *orientale* leaf extracts was dissolved in distilled water to produce a series of concentration solutions, including 100 mg/mL, 75 mg/mL, 50 mg/mL, 25 mg/mL and 12 mg/mL [70]–[73]. To examine *in vivo* effect of *P*. *orientale* leaf extracts, healthy potatoes without physical injuries or infections were scraped (1 mm deep and 8 mm wide) with a sterile nail. Then, 50 µL of 100 mg/mL, 75 mg/mL, 50 mg/mL, 25 mg/mL and 12 mg/mL solution or 50 µL of sterile distilled water (control) was put into each hole (one potato contained three holes). After 24 h, 10 µL of *C*. *michiganense* subsp. *sepedonicum* at 10^6^ CFU/mL was put into each hole. The treated potatoes were put in trays covered with plastic bags to maintain a relative humidity of approximately 95%, then incubated at 28°C. Protection percentage was calculated on the fourth day after inoculation using the following formula:
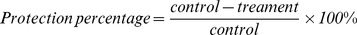



Control : The number of potatoes showing disease symptoms, treated with distilled water.

Treatment: The number of potatoes showing disease symptoms, treated with *P*. *orientale* leaf extracts.

Each experiment was repeated four times, with 60 potatoes per experiment.

### Field Experiments

Based on the results of *in vivo* assays, the dried powder of *P*. *orientale* leaf extracts was dissolved in distilled water to produce 50 mg/mL, 25 mg/mL and 12 mg/mL solutions. Overnight culture of *C*. *michiganense* subsp. *sepedonicum* was adjusted to 10^6^ CFU/mL, and was then incubated 20 µL in the tuber. When the first symptoms of bacterial ring rot of potato occurred naturally, tubers were soaked in water solutions of *P*. *orientale* extracts at 50 mg/mL, 25 mg/mL and 12 mg/mL concentration, respectively, for 15 mins. Untreated potato tubers and potato tubers treated with 50 mg/L of copper sulfate (chemical bactericides) were used as controls. Each treatment consisted of three replicates with 50 tubers each. A total of 150 tubers were treated in each variant. All field experiments were conducted on the farm of Shanxi Academy of Agricultural Science, Shanxi Province, China, during the 2010 and 2011 two growing seasons. The land accessed is not privately owned or protected. The treatments were arranged in a complete randomized block design with three plots as replicates. Ten tubers per line (distances between plants: 30 cm, and distances between lines: 40 cm) were sown and five lines per plot (4 m×3 m) were maintained. The observation of growth parameters (whole plant length, shoot length, root length, plant fresh weight, shoot fresh weight, root fresh weight, dry weight, and number of leaves) were recorded and analyzed after 8 weeks from date of planting, in both seasons.

### Solvent Partition of *P. orientale* Extracts

100 g of dried powder of *P*. *orientale* extracts was taken and mixed with 500 mL of sterile water. Then the sample was sequentially extracted using petroleum ether, chloroform, ethyl acetate, and *n*-butyl alcohol as extraction solvents (1∶1, v/v). Each partitioned extract was then concentrated to dryness. The process was repeated four times for each of the four solvents [74]. Then each dried powder from all four partitioned extracts and water partition was dissolved in DMSO to give a final concentration of 1 mg/mL for antibacterial activity assays (ADM).

### Transmission Electron Microscopy (TEM)

TEM technique was used to observe the structural changes in *C. michiganense* subsp. *sepedonicum* (ATCC 33113) induced by petroleum ether fractionation of *P*. *oriental* extracts. Logarithmic phase cells of *C. michiganense* subsp. *sepedonicum* (each approximately 10^9^ CFU/L) were treated with petroleum ether fractionation of *P*. *oriental* extracts at 0.05 mg/mL for 8 h. No treatment with petroleum ether fractionation of *P*. *oriental* extracts was as control. Cells were then collected by centrifugation and washed with 0.05 mol/L phosphate buffer saline (PBS), pH7.0. The samples were transferred to fresh 0.5% glutaraldehyde, and kept for 30 min at 4°C, centrifuged at 13,000 rpm, and fixed in 3% glutaraldehyde. Cells were further fixed in 1% OSO_4_, dehydrated in gradually increased acetone solutions, and embedded in Epon812. Ultrathin sections were cut and stained with uranyl acetate and lead citrate. Electron micrographs were taken with a JEM–1011(Tokio, Japan) transmission electron microscope at 80 kV.

### Statistical Analysis

ANOVA and *t*-test were performed on the data, using the SPSS package software (Version 17.0).
